# Enhancing nutritional quality in plants using complementary peptide for sustainable agriculture

**DOI:** 10.1093/plphys/kiae386

**Published:** 2024-07-23

**Authors:** Ashish Sharma, Anwesha Anyatama, Himanshi Gautam, Subhash Reddy Gaddam, Deeksha Singh, Hiteshwari Sinha, Prabodh Kumar Trivedi

**Affiliations:** Plant Biotechnology Division, CSIR-Central Institute of Medicinal and Aromatic Plants (CSIR-CIMAP), P.O. CIMAP, Near Kukrail Picnic Spot, Lucknow 226 015, India; Academy of Scientific and Innovative Research (AcSIR), Ghaziabad 201002, India; Plant Biotechnology Division, CSIR-Central Institute of Medicinal and Aromatic Plants (CSIR-CIMAP), P.O. CIMAP, Near Kukrail Picnic Spot, Lucknow 226 015, India; Academy of Scientific and Innovative Research (AcSIR), Ghaziabad 201002, India; Molecular Biology and Biotechnology Division, CSIR-National Botanical Research Institute (CSIR-NBRI), Rana Pratap Marg, Lucknow 226001, India; Plant Biotechnology Division, CSIR-Central Institute of Medicinal and Aromatic Plants (CSIR-CIMAP), P.O. CIMAP, Near Kukrail Picnic Spot, Lucknow 226 015, India; Plant Biotechnology Division, CSIR-Central Institute of Medicinal and Aromatic Plants (CSIR-CIMAP), P.O. CIMAP, Near Kukrail Picnic Spot, Lucknow 226 015, India; Academy of Scientific and Innovative Research (AcSIR), Ghaziabad 201002, India; Plant Biotechnology Division, CSIR-Central Institute of Medicinal and Aromatic Plants (CSIR-CIMAP), P.O. CIMAP, Near Kukrail Picnic Spot, Lucknow 226 015, India; Academy of Scientific and Innovative Research (AcSIR), Ghaziabad 201002, India; Plant Biotechnology Division, CSIR-Central Institute of Medicinal and Aromatic Plants (CSIR-CIMAP), P.O. CIMAP, Near Kukrail Picnic Spot, Lucknow 226 015, India; Academy of Scientific and Innovative Research (AcSIR), Ghaziabad 201002, India; Molecular Biology and Biotechnology Division, CSIR-National Botanical Research Institute (CSIR-NBRI), Rana Pratap Marg, Lucknow 226001, India

Dear Editor,

The demand for nutritional quality and composition is universal for a sustainable and wholesome diet. With a growing concern regarding the nutritional content of plant-based foods, individuals are turning toward animal-based products to fulfill their dietary requirements. Numerous approaches have been employed to enhance nutritional quality and yield, yet many of these face limitations (Mipeshwaree et al. 2023). Genome editing emerges as an effective strategy to improve the nutritional profile of crops (Li et al. 2022). However, a global limitation exists concerning the acceptance of CRISPR-Cas9–based edited crops (Ahmad et al. 2021). Due to regulations imposed by governing bodies focusing on food safety and security, these approaches are not directly accessible to farmers. A recent report introduced a novel class of peptides known as complementary peptides (cPEPs). cPEPs can enhance the abundance of target proteins by augmenting the recruitment of ribosomal machinery (Ormancey et al. 2023). Expanding on this finding, we proposed a hypothesis: considering the presence of conserved domains across the plant kingdom, could we develop a single cPEP for a gene containing such a conserved domain in various plant species, and would it demonstrate efficacy across different plants?

To test this hypothesis, we focused on the phenylpropanoid pathway, a pathway renowned for synthesizing secondary metabolites such as flavonols, anthocyanin, and lignin (Supplementary Fig. S1; Gautam et al. 2023). In this study, we chose the key regulators of the phenylpropanoid pathway genes to design cPEPs (Fig. 1, A to D; Supplementary Fig. S2). Specifically, two transcription factors, MYB12 and HY5, along with early and pivotal biosynthesis genes of the pathway, such as CHS, were selected for the investigation. MYB12 and HY5 act as central regulators, positively influencing several genes in this pathway (Bhatia et al. 2018). To elucidate the biological function of cPEPs (cPEPs of MYB12, CHS, and HY5), we conducted a peptide assay. We examined the protein accumulation of the corresponding genes in Arabidopsis, tobacco, and tomato after supplementation with cPEPs. Western blot analysis revealed a significantly enhanced protein level of the targeted genes in Arabidopsis, tobacco, and tomato (Fig. 1, E to M). Increased protein levels of MYB12, CHS, and HY5 are known to influence the expression of other pathway genes (Gangappa and Botto 2016). We did not observe any significant modulation at both the transcript and protein levels of the target genes (HY5 and MYB12 in case of cPEPCHS; HY5 in case of cPEPMYB12). However, genes regulated by MYB12 or HY5 were significantly enhanced (Fig. 1, N to P; Supplementary Figs. S3 to S5). Together, these results suggest that the exogenous application of cPEPs has great potential to modulate the expression of target and associated genes.

**Figure 1.**
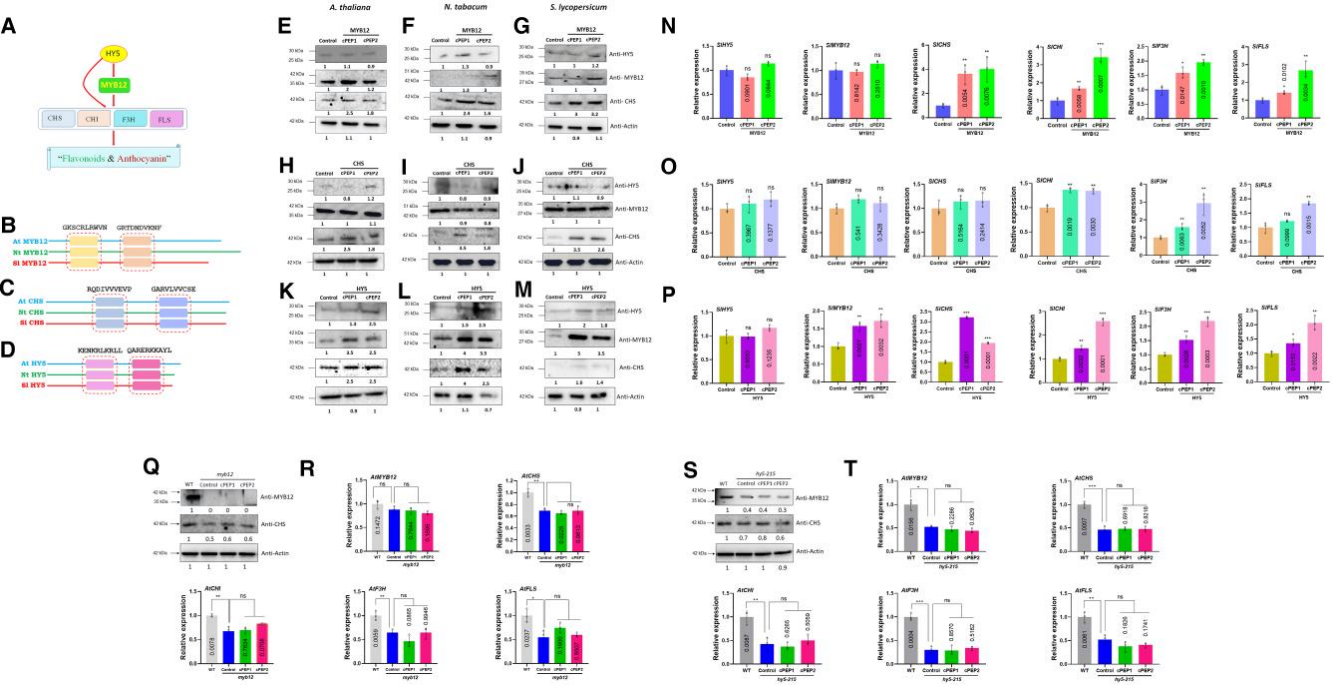
Regulation of target and associated genes by exogenous cPEPs (MYB12, CHS, and HY5). **A)** A model for transcriptional regulation of CHS, CHI, F3H, and FLS by HY5 directly and via MYB12. HY5, a bZIP transcription factor, binds to promoters of MYB12, F3H, and FLS and positively regulates their transcription. Similarly, MYB12, an R2R3-MYB transcription factor, acts as a transcriptional activator of CHS, CHI, F3H, and FLS. **B to D)** Schematic structure of *Arabidopsis thaliana*, *Nicotiana tabacum*, and *Solanum lycopersicum* protein sequence represented by straight line of MYB12, CHS, and HY5. Color boxes show conserved amino acid sequences chosen for designing cPEP. **E to G)** Western blot analysis of HY5, MYB12, and CHS protein in seedlings of WT *Arabidopsis thaliana***E)**, *Nicotiana tabacum***F)**, and *Solanum lycopersicum***G)** grown on half-strength MS medium for 5 or 10 d and then dipped in liquid half-strength MS medium supplemented with water (control), 50 *µ*M cPEP1MYB12, and cPEP2MYB12 for 48 h. Actin was used as the loading control. **H to J)** Western blot analysis of HY5, MYB12, and CHS protein in seedlings of WT *Arabidopsis thaliana***H)**, *Nicotiana tabacum***I)**, and *Solanum lycopersicum***J)** grown on half-strength MS medium for 5 or 10 d and then dipped in liquid half-strength MS medium supplemented with water (control), 50 *µ*M cPEP1CHS, and cPEP2CHS for 48 h. Actin was used as the loading control. **K to M)** Western blot analysis of HY5, MYB12, and CHS protein in seedlings of WT *Arabidopsis thaliana***K)**, *Nicotiana tabacum***L)**, and *Solanum lycopersicum***M)** grown on half-strength MS medium for 5 or 10 d and then dipped in liquid half-strength MS medium supplemented with water (control), 50 *µ*M cPEP1HY5, and cPEP2HY5 for 48 h. Actin was used as the loading control. **N)** Expression analysis of phenylpropanoid pathway genes *SlHY5, SlMYB12, SlCHS, SlCHI, SlFLS*, and *SlF3H* in seedlings of *Solanum lycopersicum* (cv. Micro Tom) grown on half-strength MS medium for 10 d and then dipped in liquid half-strength MS medium supplemented with water (control), 50 *µ*M cPEP1MYB12, and cPEP2MYB12 for 48 h. **O)** Expression analysis of phenylpropanoid pathway genes *SlHY5, SlMYB12, SlCHS, SlCHI, SlFLS5,* and *SlF3H* in seedlings of *Solanum lycopersicum* (cv. Micro Tom) grown on half-strength MS medium for 10 d and then dipped in liquid half-strength MS medium supplemented with water (control), 50 *µ*M cPEP1CHS, and cPEP2CHS for 48 h. **P)** Quantification of phenylpropanoid pathway genes *SlHY5, SlMYB12, SlCHS, SlCHI, SlFLS*, and *SlF3H* in seedlings of *Solanum lycopersicum* (cv. Micro Tom) grown on half-strength MS medium for 10 d and then dipped in liquid half-strength MS medium supplemented with water (control), 50 *µ*M cPEP1HY5, and cPEP2HY5 for 48 h. **Q to T)** Western blot analysis of MYB12 and CHS protein in *myb12***Q)** and *hy5-215***S)** mutant seedlings of A*rabidopsis thaliana* grown on half-strength MS medium for 5 d and then dipped in liquid half-strength MS medium supplemented with water (control), 50 *µ*M cPEP1MYB12, cPEP2MYB12, and 50 *µ*M cPEP1HY5, cPEP2HY5 for 48 h, respectively. Expression analysis of phenylpropanoid pathway genes *AtMYB12*, *AtCHS*, *AtCHI*, *AtF3H, AtFLS* in *myb12***R)** and *hy5-215***T)** mutant seedlings of *Arabidopsis thaliana* grown on half-strength MS medium for 5-days and then dipped in liquid half-strength MS medium supplemented with water (control), 50 *µ*M cPEP1MYB12, cPEP2MYB12 and 50 *µ*M cPEP1HY5, cPEP2HY5 for 48 h, respectively. Actin was used as the loading control. Values above and below panel in western blot show the quantification of each band with respect to the loading control.

Next, we investigated the necessity of functional mRNA for cPEP action by employing various mutants (*myb12* and *hy5-215*) and genome-edited lines of tobacco and tomato (*NtHY5^CR^* and *SlHY5^CR^*; Singh et al. 2024; Sinha et al. 2024) to assess the responsiveness of cPEPs. Western blot and expression analysis results indicated that no significant alteration in the protein accumulation and expression was observed in mutant and edited lines supplemented with cPEPs (Fig. 1, Q to T; Supplementary Figs. S6 and S7). Based on the results, we concluded that functional mRNA is essential for cPEP function. To provide additional evidence highlighting the significance of cPEP, we conducted a detailed promoter reporter assay to examine the responsiveness toward cPEPs. A peptide assay was conducted using various promoter–reporter lines. The histochemical assay, GUS gene expression, and endogenous protein level clearly revealed enhanced GUS activity in seedlings treated with cPEPMYB12 and cPEPHY5 (Supplementary Fig. S8).

Diphenylboric acid-2-aminoethyl ester (DPBA) staining was employed to assess plant flavonol accumulation. Confocal images revealed enhanced accumulation of kaempferol (yellow fluorescence) and quercetin (green fluorescence) in the root elongation zone of seedlings grown on media supplemented with cPEPMYB12 compared to control (Fig. 2A, Supplementary Fig. S9 and S10). Quantification of flavonol aglycones (kaempferol and quercetin) provided further evidence that cPEPMYB12 promotes the accumulation of flavonols (Fig. 2, B and C; Supplementary Fig. S9 and S10). Enhanced flavonols inhibit auxin transport, leading to shorter root length (Brown et al. 2001). Analysis of root length data indicated a significant reduction in the root length of seedlings grown on media with cPEPMYB12 (Supplementary Fig. S11, A to D). Enhanced accumulation of late biosynthetic genes in the phenylpropanoid pathway redirects pathway flux toward flavonols and anthocyanins, ultimately resulting in lower lignin accumulation in plants (Sharma et al. 2020). Decreased lignin accumulation was observed in cPEP-supplemented seedlings compared to those grown on control media (Supplementary Fig. S11E). Anthocyanin quantification indicated that cPEP-treated seedlings of *Arabidopsis*, tobacco, and tomato accumulated elevated levels of anthocyanin (Fig. 2D; Supplementary Figs. S12 to S14). Expression analysis of anthocyanin biosynthesis genes revealed a significant increase in transcript levels in seedlings supplemented with cPEPs (Supplementary Figs. S12 to S14).

**Figure 2.**
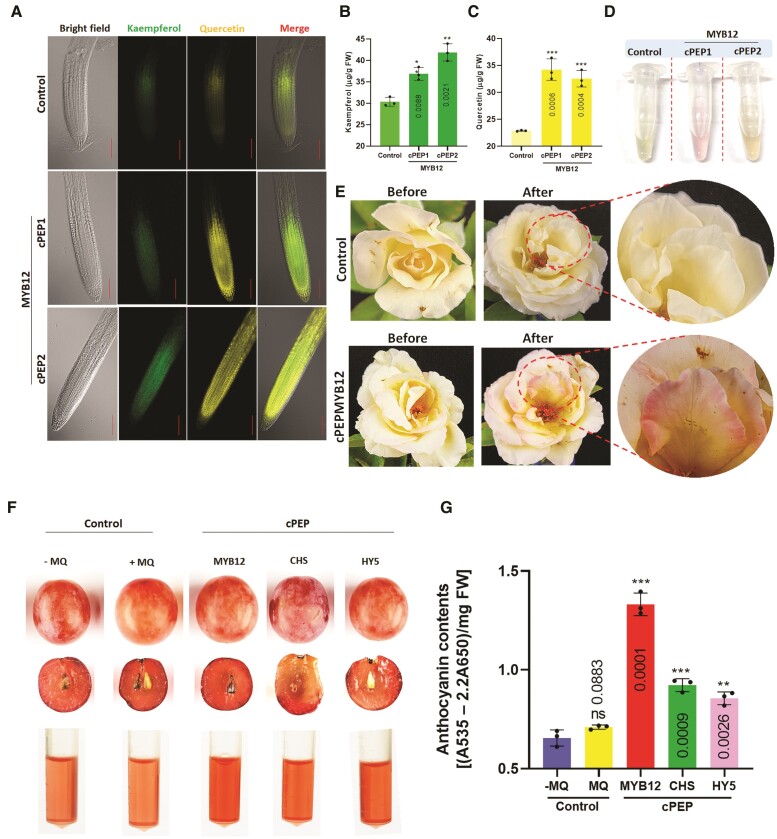
Application of cPEPs (MYB12, CHS, and HY5) enhances flavonols and anthocyanin. **A)** Confocal images of DPBA staining of roots of 3-d-old *Arabidopsis* seedlings grown on half-strength MS medium supplemented with water (control), 50 *µ*M cPEP1MYB12, and cPEP2MYB12. **B and C)** Quantification of kaempferol and quercetin content in 5-d-old WT seedlings of *Arabidopsis* grown on half-strength MS medium supplemented with water (control), 50 *µ*M cPEP1MYB12, and cPEP2MYB12. **D)** Representative image of anthocyanin accumulation in 5-d-old WT seedlings of *Arabidopsis* grown on half-strength MS medium supplemented with water (control), 50 *µ*M cPEP1MYB12, and cPEP2MYB12. **E)** Representative image of rose flower sprayed with liquid half-strength MS medium supplemented with water (control) and 50 *µ*M cPEPMYB12, respectively. **F)** Representative image of grapes and its anthocyanin accumulation without any spray (−MQ), when sprayed with liquid half-strength MS medium supplemented with water (+MQ), 50 *µ*M cPEPMYB12, cPEPCHS, and cPEPHY5 respectively. **G)** Quantification of anthocyanin in grapes without any spray (−MQ), when sprayed with liquid half-strength MS medium supplemented with water (+MQ), 50 *µ*M cPEPMYB12, cPEPCHS, and cPEPHY5, respectively.

To demonstrate the universality of cPEP action, we used *Rosa* sp., a significant botanical species known for its economic and ornamental values. Rose petals exhibit various colors due to difference in its anthocyanin content (Yan et al. 2023). We found it intriguing to explore whether the exogenous application of cPEPs can alter the color of rose petals, given that roses also possess similar cPEP sequences (Supplementary Fig. S2). When sprayed with cPEP exogenously, the white or light yellow–colored rose petals developed pink/red patches, while the control treatment (sprayed only with water) showed no color variation (Fig. 2E; Supplementary Fig. S15). For further validation, total anthocyanin quantification was done, and the result indicated significant enhancement in anthocyanin content in rose petals sprayed with cPEPs (Supplementary Fig. S15, C and D). Anthocyanins, known to contribute toward the red pigmentation of grapes (*Vitis vinifera*), are associated with various health benefits (He et al. 2010).

Our examination of the amino acid sequences in grapes revealed a similar sequence of cPEPs in grapes as found in other plants in this study (Supplementary Fig. S2). A significant increase in pigmentation was observed in the epicarp and mesocarp regions of cPEP-supplemented grapes compared to controls (Fig. 2, F and G). These results suggest that the exogenous application of cPEPs can increase anthocyanin content, influencing the coloring of rose petals and grapes. Our results suggest that the design of a single conserved cPEP can enhance level of specific protein across species. In conclusion, our study illustrates how the application of cPEPs derived from key regulatory (HY5 and MYB12) and structural genes (CHS) in the phenylpropanoid pathway can bolster targeted protein translation. This modulation leads to increased accumulation of essential flavonols and anthocyanins, known for their nutritive value, in diverse plant species, including Arabidopsis, tobacco, tomato, rose, and grapes. By doing so, our approach presents a promising strategy for elevating both nutritional quality and economic viability, offering an alternative to reliance solely on biotechnological interventions in agriculture.

## Supplementary Material

kiae386_Supplementary_Data

## Supplementary data

The following materials are available in the online version of this article.

**Supplementary Figure S1.** Phenylpropanoid pathway.

**Supplementary Figure S2.** Alignment of Arabidopsis, tobacco, tomato, rose, and grape MYB12, CHS, and HY5 amino acid sequence and cPEP design.

**Supplementary Figure S3.** Effect of cPEPMYB12 on phenylpropanoid pathway genes in Arabidopsis and tobacco.

**Supplementary Figure S4.** Effect of cPEPCHS on phenylpropanoid pathway genes in Arabidopsis and tobacco.

**Supplementary Figure S5.** Effect of cPEPHY5 on phenylpropanoid pathway genes in Arabidopsis and tobacco.

**Supplementary Figure S6.** Functional mRNA is required for cPEP action in tobacco.

**Supplementary Figure S7.** Functional mRNA is required for cPEP action in tomato.

**Supplementary Figure S8.** Transcriptional regulation of FLS, CHS, and miPEP858a by cPEPMYB12 and cPEPHY5.

**Supplementary Figure S9.** Exogenous application of cPEPs enhances flavonol accumulation in tobacco.

**Supplementary Figure S10.** Exogenous application of cPEPs enhances flavonol accumulation in tomato.

**Supplementary Figure S11.** Enhanced expression of pathway gene diverts the flux toward flavonol.

**Supplementary Figure S12.** Exogenous application of cPEPs (MYB12, CHS, and HY5) enhances anthocyanin and its biosynthesis genes in Arabidopsis.

**Supplementary Figure S13.** Exogenous application of cPEPs (MYB12, CHS, and HY5) enhanced anthocyanin and its biosynthesis genes in tobacco.

**Supplementary Figure S14.** Exogenous application of cPEPs (MYB12, CHS, and HY5) enhances anthocyanin and its biosynthesis genes in tomato.

**Supplementary Figure S15.** Exogenous application of cPEP (CHS and HY5) induced coloring in white/yellow rose petals.
