# Supplementary material for: Enhancing nutritional quality in plants using complementary peptide for sustainable agriculture
Source: Plant Physiol. 2024 Jul 23;196(2):711–5. doi: 10.1093/plphys/kiae386 (PMC11444269; doi:10.1093/plphys/kiae386)
Supplement: kiae386_Supplementary_Data [file kiae386_supplementary_data.pdf]

Supplemental Information

Enhancing Nutritional Quality in Plants using Complementary Peptide for Sustainable Agriculture

Ashish Sharma[1,2], Anwesha Anyatama[1], Himanshi Gautam[2,3], Subhash Reddy Gaddam[1], Deeksha Singh[1,2], Hiteshwari Sinha[1,2], Prabodh Kumar Trivedi[1,2,3,*]

[1]CSIR- Central Institute of Medicinal and Aromatic Plants (CSIR-CIMAP) P.O. CIMAP, Near Kukrail Picnic Spot, Lucknow-226 015, India

[2]Academy of Scientific and Innovative Research (AcSIR), Ghaziabad- 201002, India

[3]CSIR-National Botanical Research Institute, Council of Scientific and Industrial Research (CSIR-NBRI), Rana Pratap Marg, Lucknow-226001, India

The author responsible for the distribution of materials integral to the findings presented in this article in accordance with the policy described in the Instructions for Authors (https://academic.oup.com/plphys/pages/General-Instructions) is Prabodh K. Trivedi.

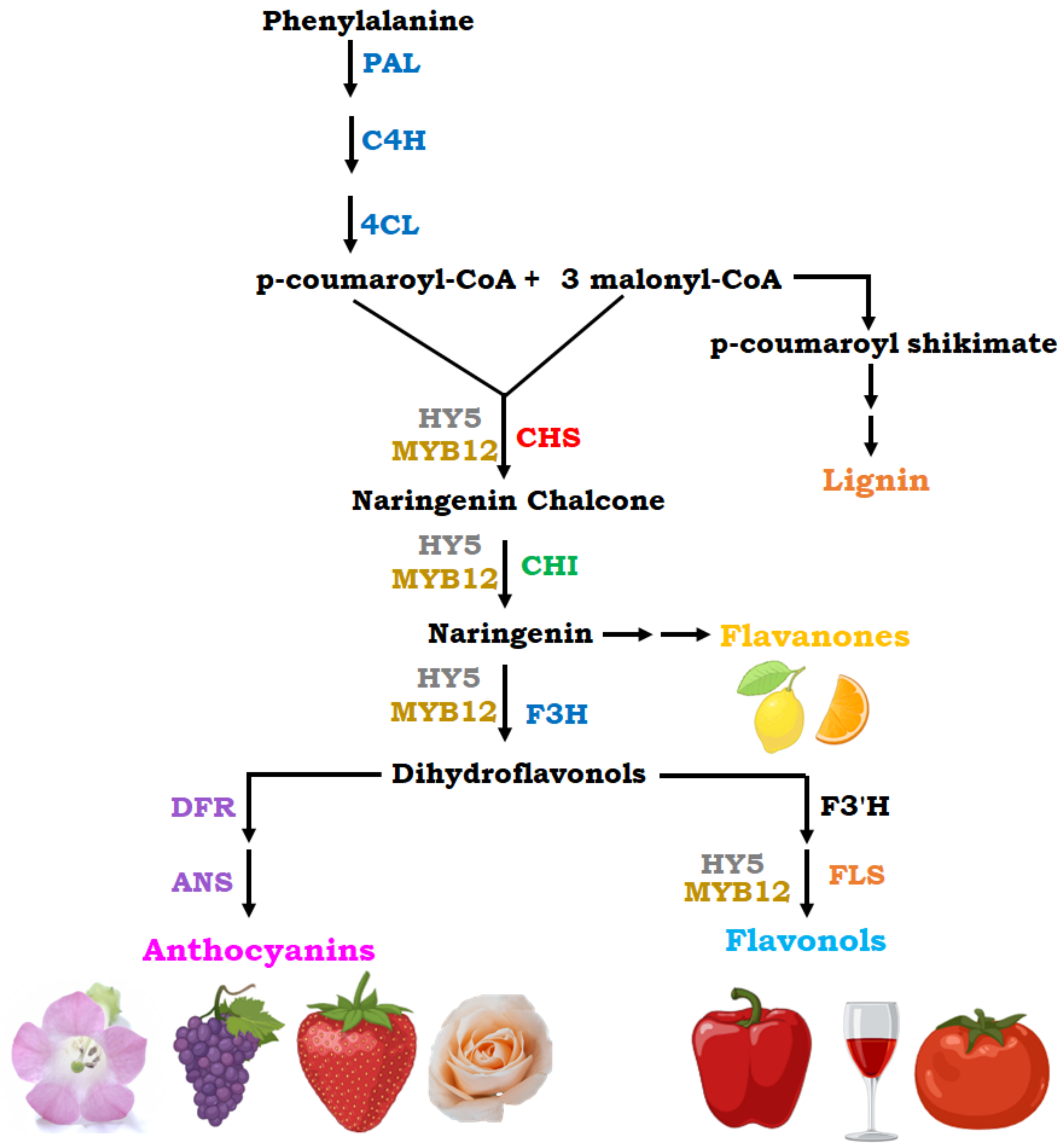

Supplemental Figure S1. Phenylpropanoid pathway. Schematic representation of phenylpropanoid pathway showing sequential enzymatic synthesis of flavonoids, anthocyanin and lignin.

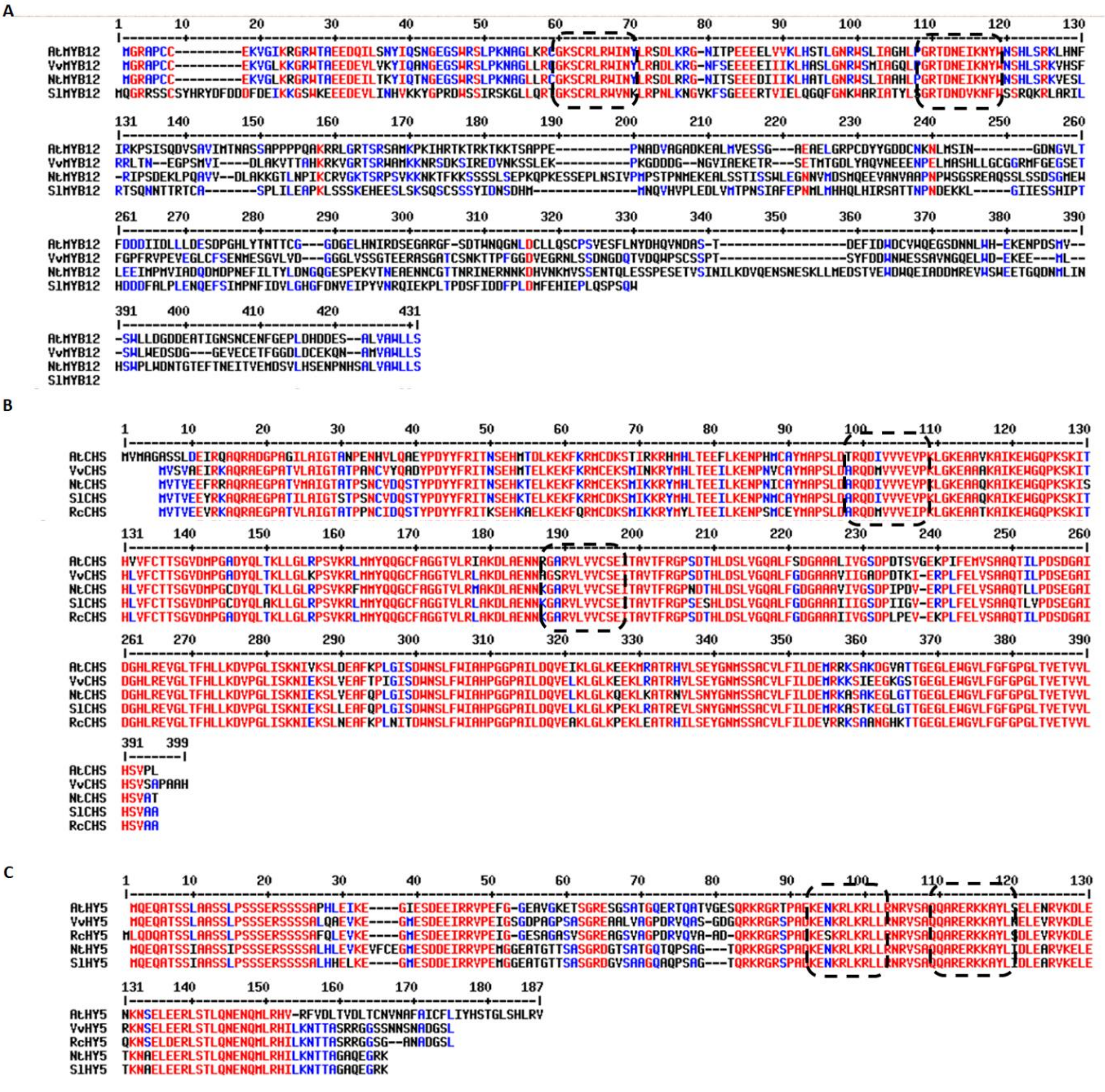

Supplemental Figure S2. Alignment of Arabidopsis, tobacco, tomato, rose and grape MYB12, CHS and HY5 amino acid sequence and cPEPs design. (A-C) Amino acid sequence alignment of *Arabidopsis thaliana* (At), *Nicotiana tabacum* (Nt), *Solanum lycopersicum* (Sl), *Rosa chinensis* (Rc) and *Vitis vinifera* (Vv) MYB12, CHS and HY5. Sequence surrounded by black-dashed square box represents common regions of each protein sequence and were used to design cPEP1 and cPEP2 of each protein respectively. Amino acids were aligned using http://multalin.toulouse.inra.fr/multalin/ online tool.

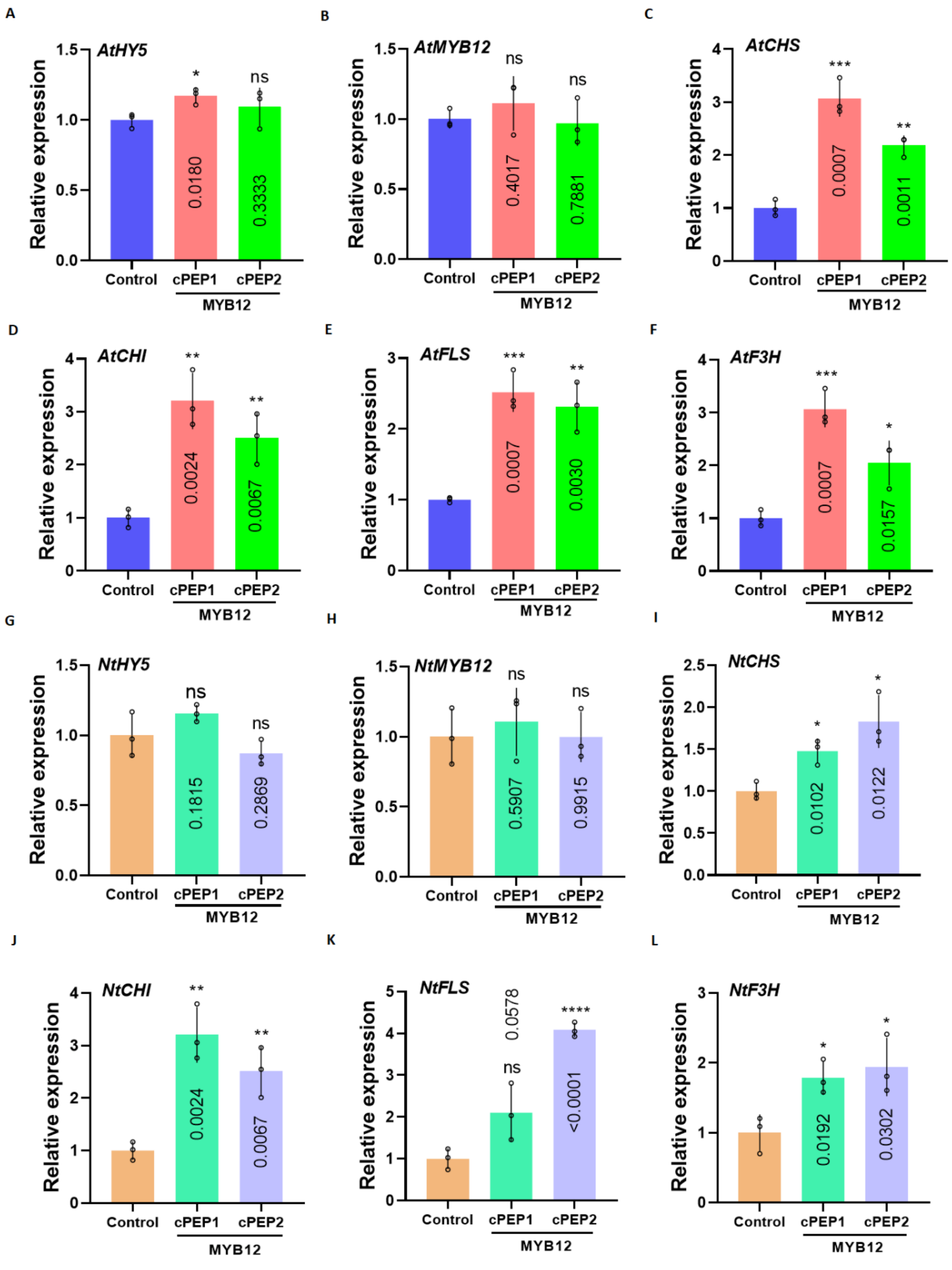

Supplemental Figure S3. Effect of cPEPMYB12 on Phenylpropanoid pathway genes in Arabidopsis and tobacco. Expression of HY5, *MYB12, CHS, CHI, FLS*, and *F3H* in seedlings of *Arabidopsis thaliana* **(A-F)** and *Nicotiana tabacum* **(G-L)** grown on half-strength MS medium for 5 and 15 days respectively, and then dipped in liquid half-strength MS medium supplemented with water (control), 0.50 µM cPEP1MYB12 and cPEP2MYB12 for 48 hours.

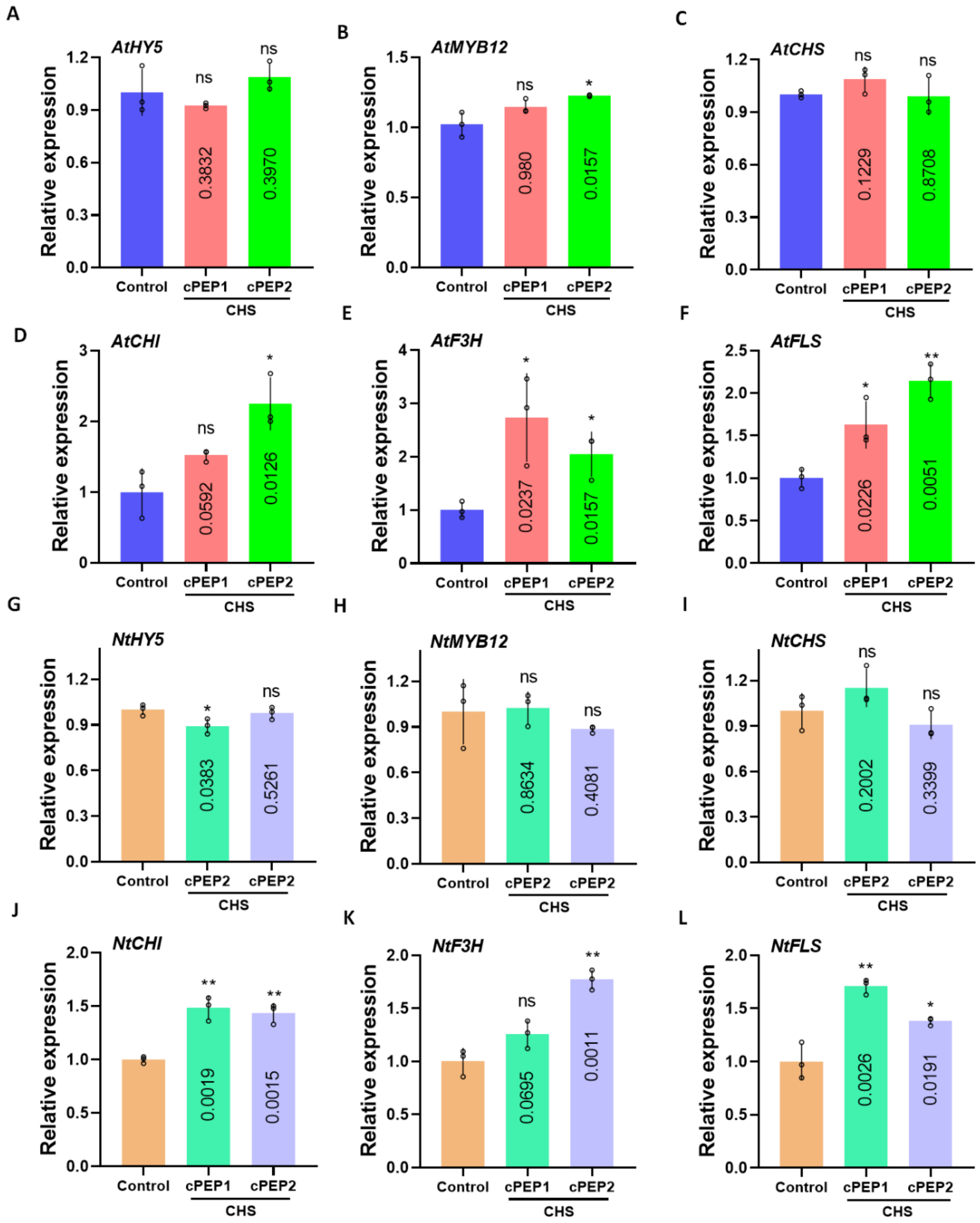

Supplemental Figure S4. Effect of cPEPCHS on Phenylpropanoid pathway genes in Arabidopsis and tobacco. Expression of *HY5*, *MYB12, CHS, CHI, FLS*, and *F3H* in seedlings of *Arabidopsis thaliana* **(A-F)** and *Nicotiana tabacum* **(G-L)** grown on half-strength MS medium for 5 and 15 days respectively, and then dipped in liquid half-strength MS medium supplemented with water (control), 0.50 µM cPEP1CHS and cPEP2CHS for 48 hours.

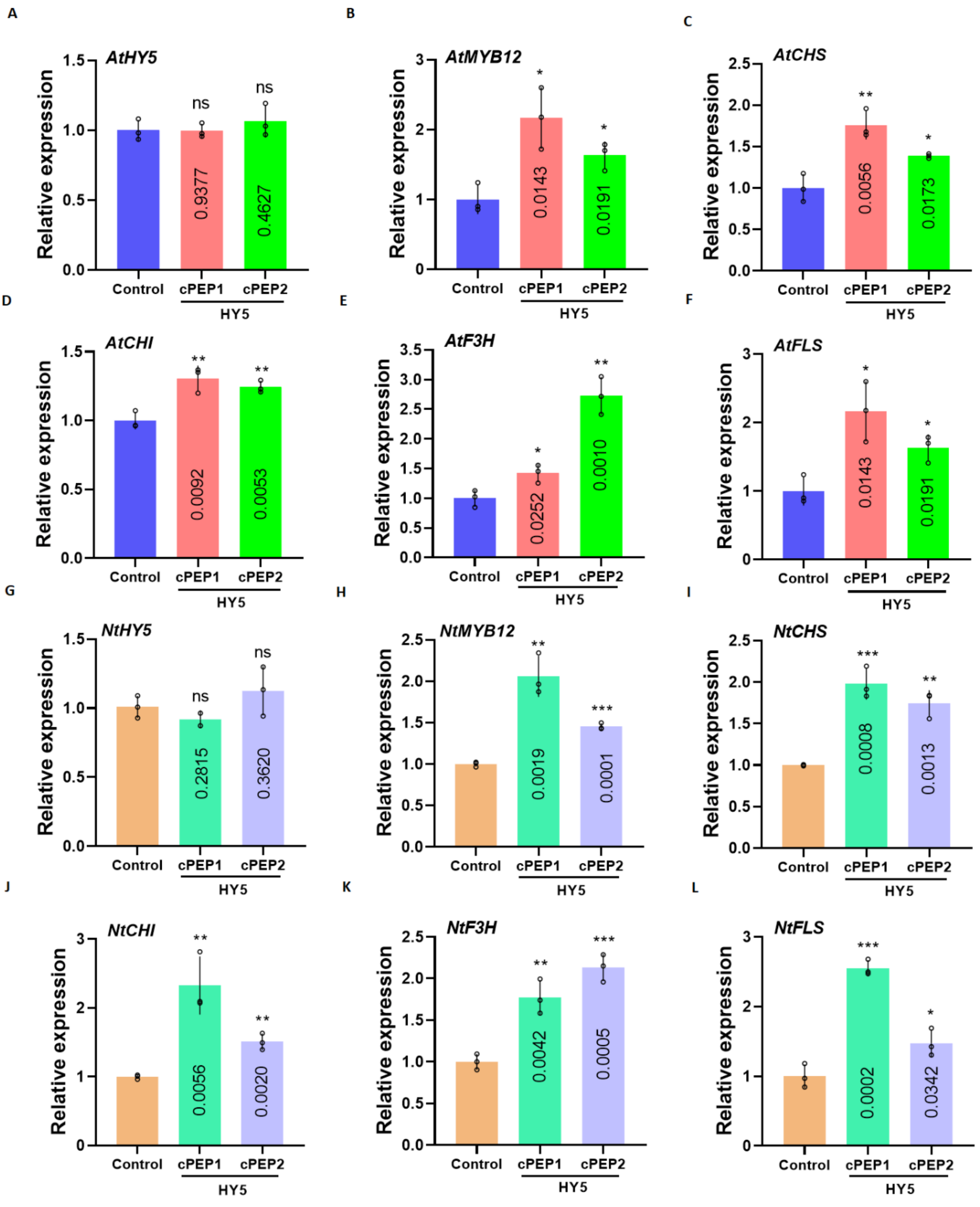

Supplemental Figure S5. Effect of cPEPHY5 on Phenylpropanoid pathway genes in Arabidopsis and tobacco. Expression of *HY5*, *MYB12, CHS, CHI, FLS*, and *F3H* in seedlings of *Arabidopsis thaliana* **(A-F)** and *Nicotiana tabacum* **(G-L)** grown on half-strength MS medium for 5 and 15 days respectively, and then dipped in liquid half-strength MS medium supplemented with water (control), 0.50 µM cPEP1HY5 and cPEP2HY5 for 48 hours.

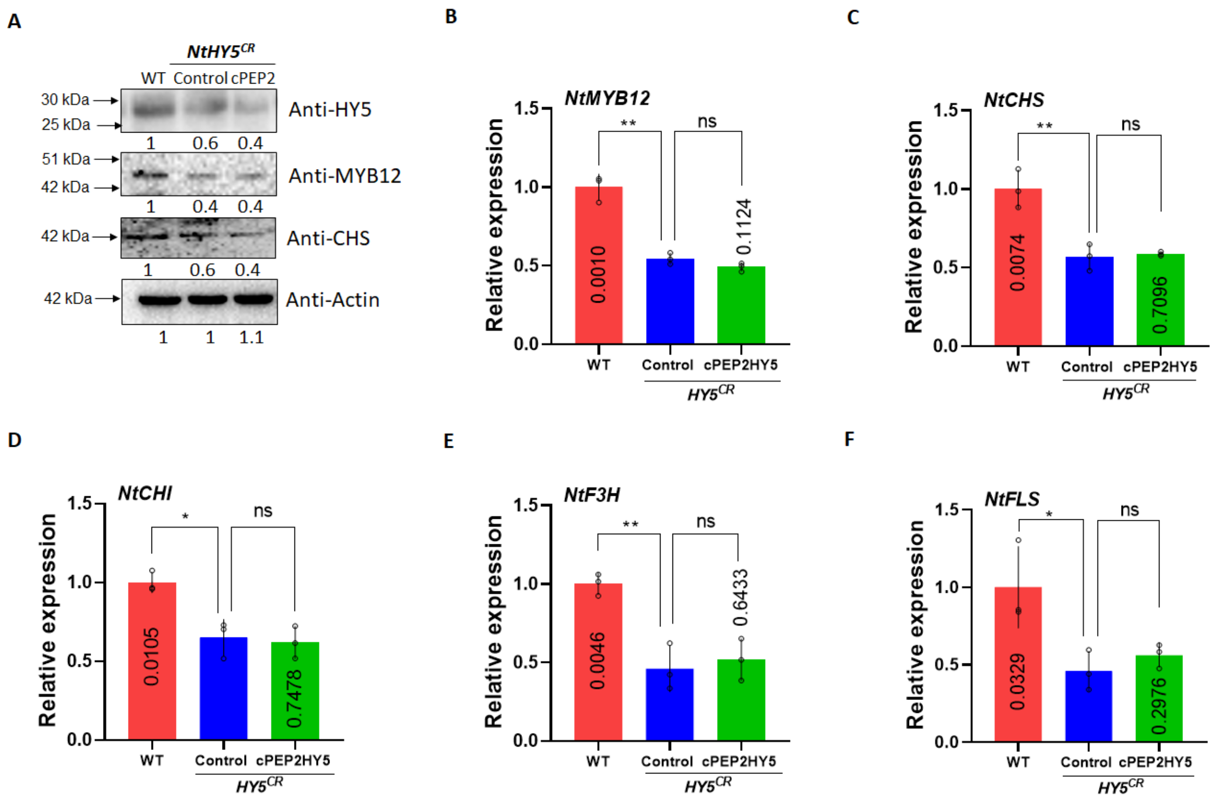

Supplemental Figure S6. Functional mRNA is required for cPEP action in tobacco. (A) Western blot analysis of HY5, MYB12 and CHS protein in *Nicotiana tabacum* seedlings of WT and *HY5*^{CR} edited line grown on half-strength MS medium for 15-days and then dipped in liquid half-strength MS medium supplemented with water (control) and 0.50 µM cPEP2HY5 for 48 hours. Actin was used as the loading control. Values in upper and lower panel in western blot show the quantification of each band with respect to the loading control. **(B-F)** Expression of phenylpropanoid pathway genes *NtMYB12, NtCHS, NtCHI, NtF3H* and *NtFLS* in *Nicotiana tabacum* seedlings of WT and *HY5*^{CR} edited line grown on half-strength MS medium for 15-days and then dipped in liquid half-strength MS medium supplemented with water (control) and 0.50 µM cPEP2HY5 for 48 hours.

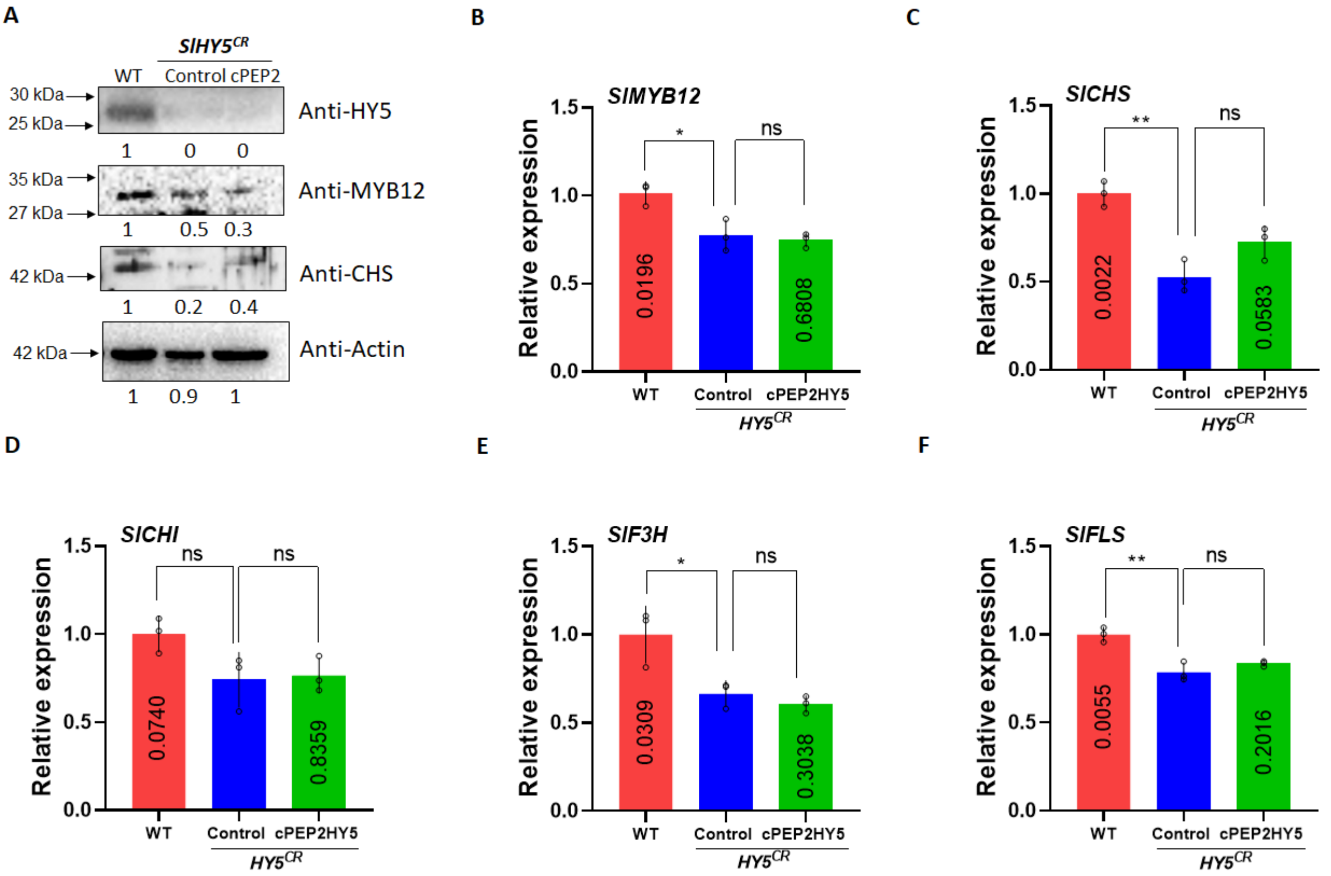

Supplementary Figure S7. Functional mRNA is required for cPEP action in tomato. (A) Western blot analysis of HY5, MYB12 and CHS protein in *Solanum lycopersicum* seedlings of WT and *HY5*^{CR} edited line grown on half-strength MS medium for 10-days and then dipped in liquid half-strength MS medium supplemented with water (control) and 0.50 µM cPEP2HY5 for 48 hours. Actin was used as the loading control. Values in upper and lower panel in western blot show the quantification of each band with respect to the loading control. **(B-F)** Expression of phenylpropanoid pathway genes *SlMYB12, SlCHS, SlCHI, SlF3H* and *SlFLS* in *Solanum lycopersicum* seedlings of WT and *HY5*^{CR} edited line grown on half-strength MS medium for 10-days and then dipped in liquid half-strength MS medium supplemented with water (control) and 0.50 µM cPEP2HY5 for 48 hours.

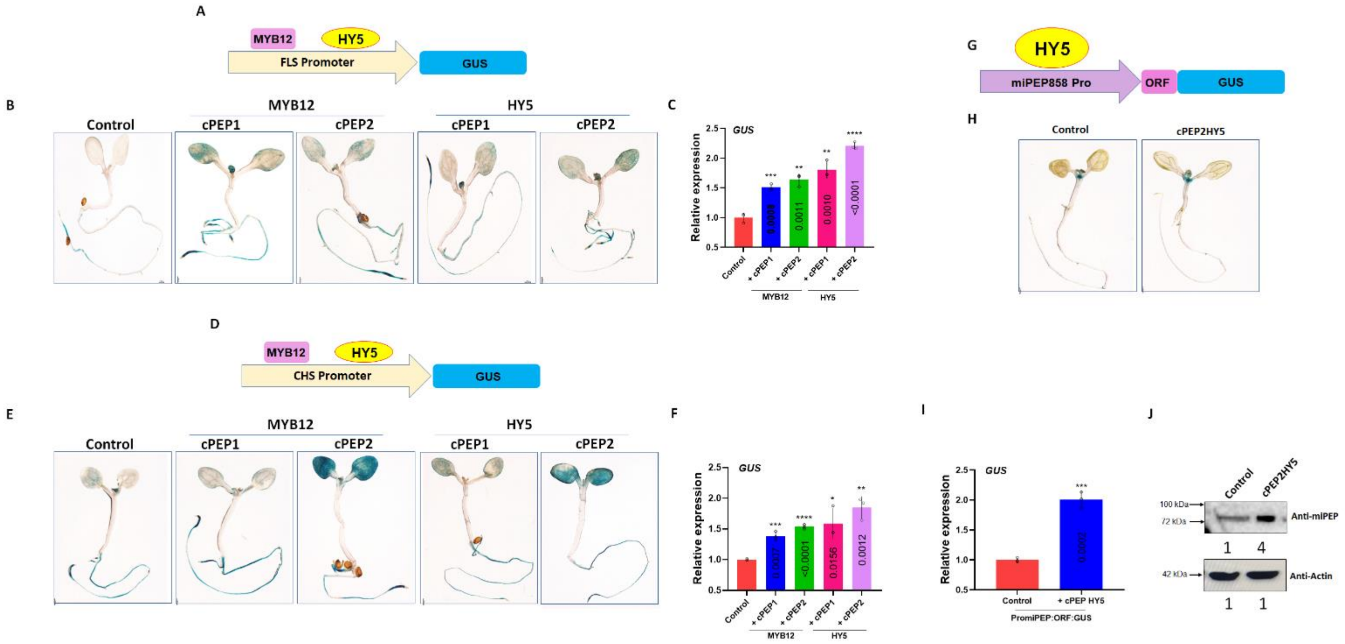

Supplementary Figure S8. Transcriptional regulation of FLS, CHS and miPEP858a by cPEPMYB12 and cPEPHY5. (**A, D)** Schematic representation of MYB12 and HY5 binding on Promoter: reporter construct of FLS and CHS Promoter respectively. (**B, E)** Histochemical staining showing GUS activity in five-day-old FLS and CHS promoter line grown on half-strength MS medium for five-days and then dipped in liquid half-strength MS medium supplemented with water (control), 0.50 µM cPEP1MYB12, cPEP2MYB12, cPEP1HY5 and cPEP2HY5 for 48 hours. (**C, F)** Relative expression of GUS in FLS and CHS Promoter line seedlings grown on half-strength MS medium for 5 days and then dipped in liquid half-strength MS medium supplemented with water (control), 0.50 µM cPEP1MYB12, cPEP2MYB12, cPEP1HY5 and cPEP2HY5 for 48 hours. (**G)** Schematic representation of HY5 binding to promoter region of Promoter: reporter construct in miPEP858 Pro:ORF::GUS line. (**H)** Histochemical staining showing GUS activity in miPEP858 Pro:ORF::GUS line grown on half-strength MS medium for 5 days and then dipped in liquid half-strength MS medium supplemented with water (control) and 0.50 µM cPEP2HY5 for 48 hours. (**I)** Relative expression of GUS in seedlings of miPEP858 Pro:ORF::GUS line grown on half-strength MS medium for 5 days and then dipped in liquid half-strength MS medium supplemented with water (control) and 0.50 µM cPEP2HY5 for 48 hours. (**J)** Western blot analysis of miPEP858a+GUS protein in seedlings of Pro:miPEP858::GUS line grown on half-strength MS medium for 5 days and then dipped in liquid half-strength MS medium supplemented with water (control) and 0.50 µM cPEP2HY5 for 48 hours. Actin was used as the loading control. Values in upper and lower panel in western blot show the quantification of each band with respect to the loading control.

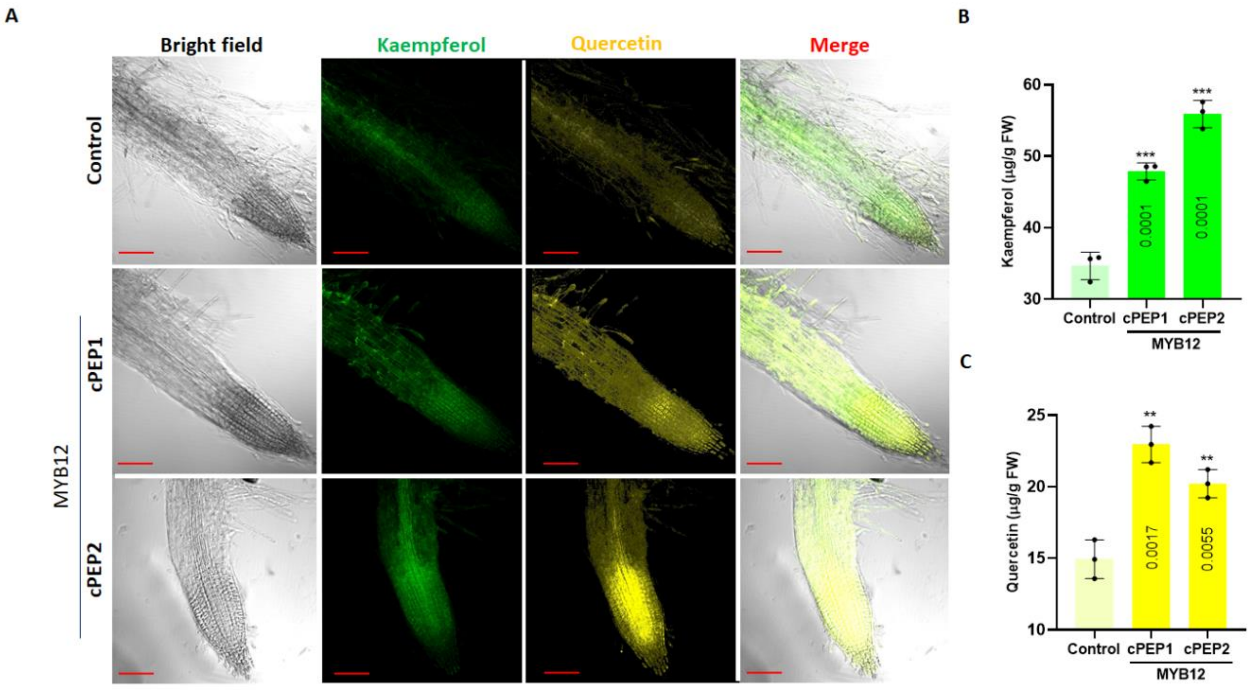

Supplementary Figure S9. Exogenous application of cPEPs enhances flavonol accumulation in tobacco. (A) Confocal images of DPBA staining of roots of 5-day old *Nicotiana tabacum* seedlings grown on half-strength MS medium supplemented with water (control), and 0.50 µM cPEP1MYB12 and cPEP2MYB12. Scale bar 100 uM. (**B, C)** Quantification of kaempferol and quercetin content in 15-day old WT seedlings of *Nicotiana tabacum* grown on half-strength MS medium supplemented with water (control), and 0.50 µM cPEP1MYB12 and cPEP2MYB12.

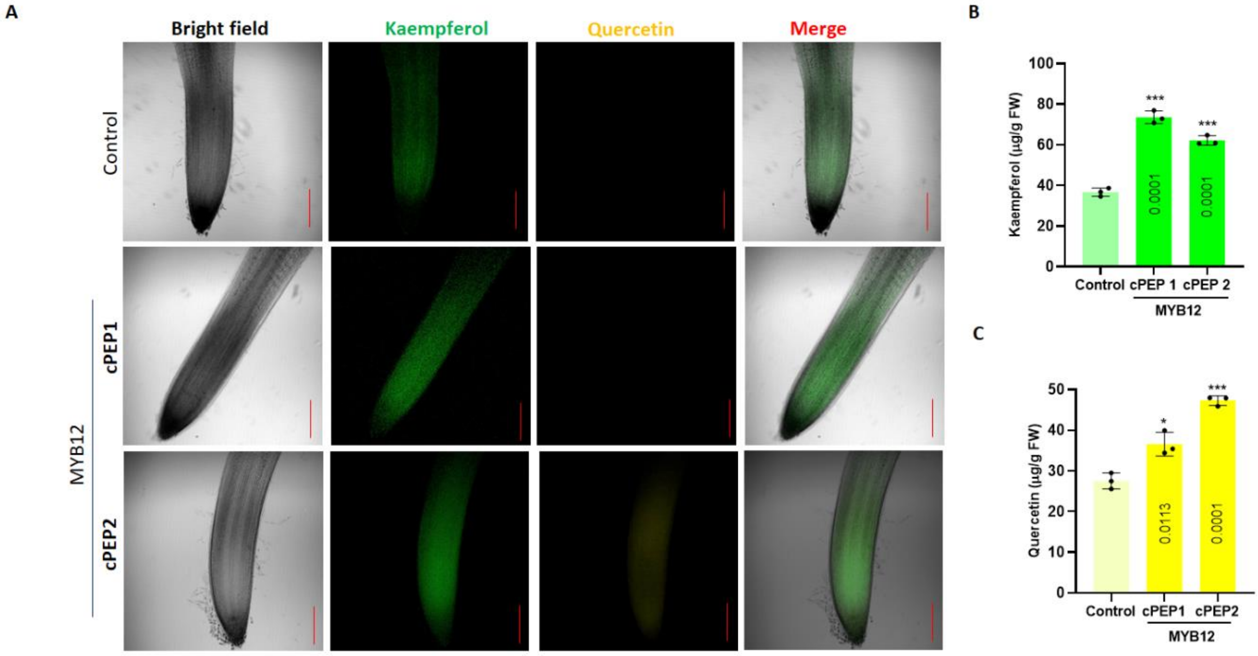

Supplemental Figure S10. Exogenous application of cPEPs enhances flavonol accumulation in tomato. (A) Confocal images of DPBA staining of roots of 5-day-old *Solanum lycopersicum* seedlings grown on half-strength MS medium supplemented with water (control), and 0.50 µM cPEP1MYB12 and cPEP2MYB12. Scale bar 100 uM. **(B, C)** Quantification of kaempferol and quercetin content in 10-day-old WT seedlings of *Solanum lycopersicum* grown on half-strength MS medium supplemented with water (control), and 0.50 µM cPEP1MYB12 and cPEP2MYB12.

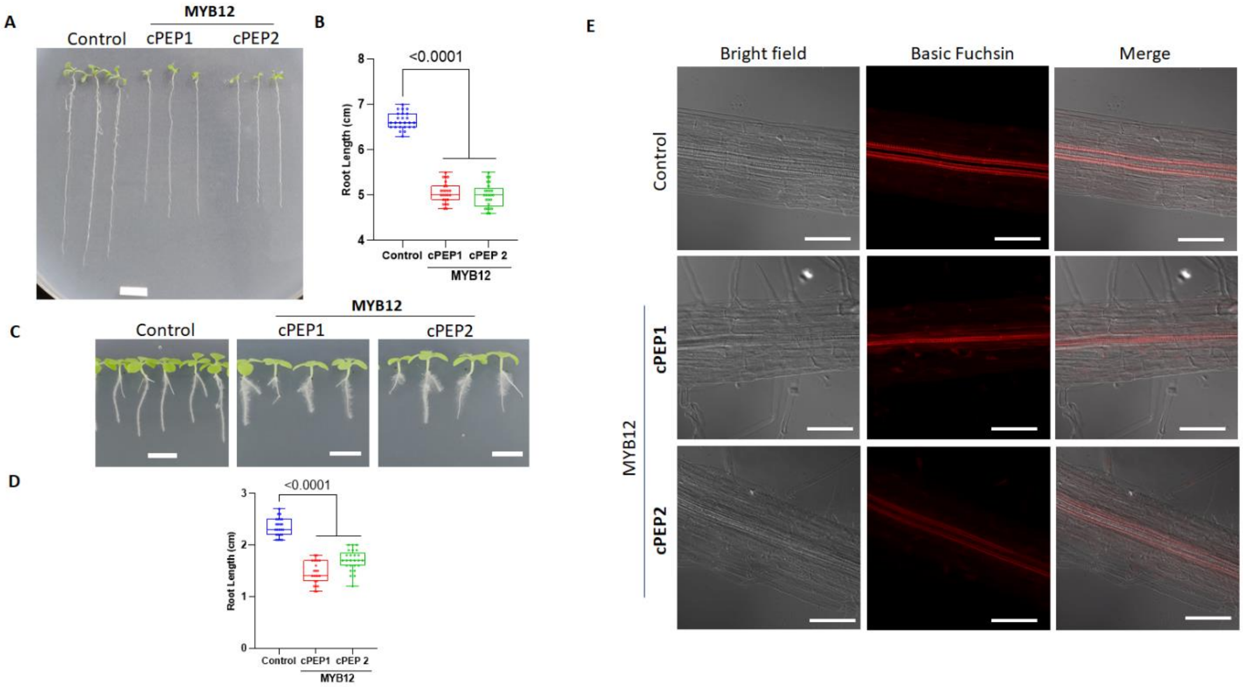

Supplemental Figure S11. Enhanced expression of pathway gene divert the flux towards flavanol. (A, C) Representative image of ten-day-old WT *Arabidopsis thaliana* and 15-day old *Nicotiana tabacum* seedlings grown on half-strength MS medium supplemented with water (control), and 0.50 µM cPEP1MYB12 and cPEP2 MYB12. Scale bar, 1 cm. (**B, D)** Root lengths of 10-day-old WT *Arabidopsis thaliana* and 15-day-old *Nicotiana tabacum* grown on half-strength MS medium supplemented with water (control), and 0.50 µM cPEP1MYB12 and cPEP2MYB12, n= 30 independent seedlings (small open circles) **(E)** 5-day-old Arabidopsis seedlings grown on half-strength MS medium supplemented with water (control), and 0.50 µM cPEP1MYB12 and cPEP2MYB12. Roots were stained with basic fuchsin to visualize lignin at 20x magnification. (Scale bar = 50 µM) in the elongation zone.

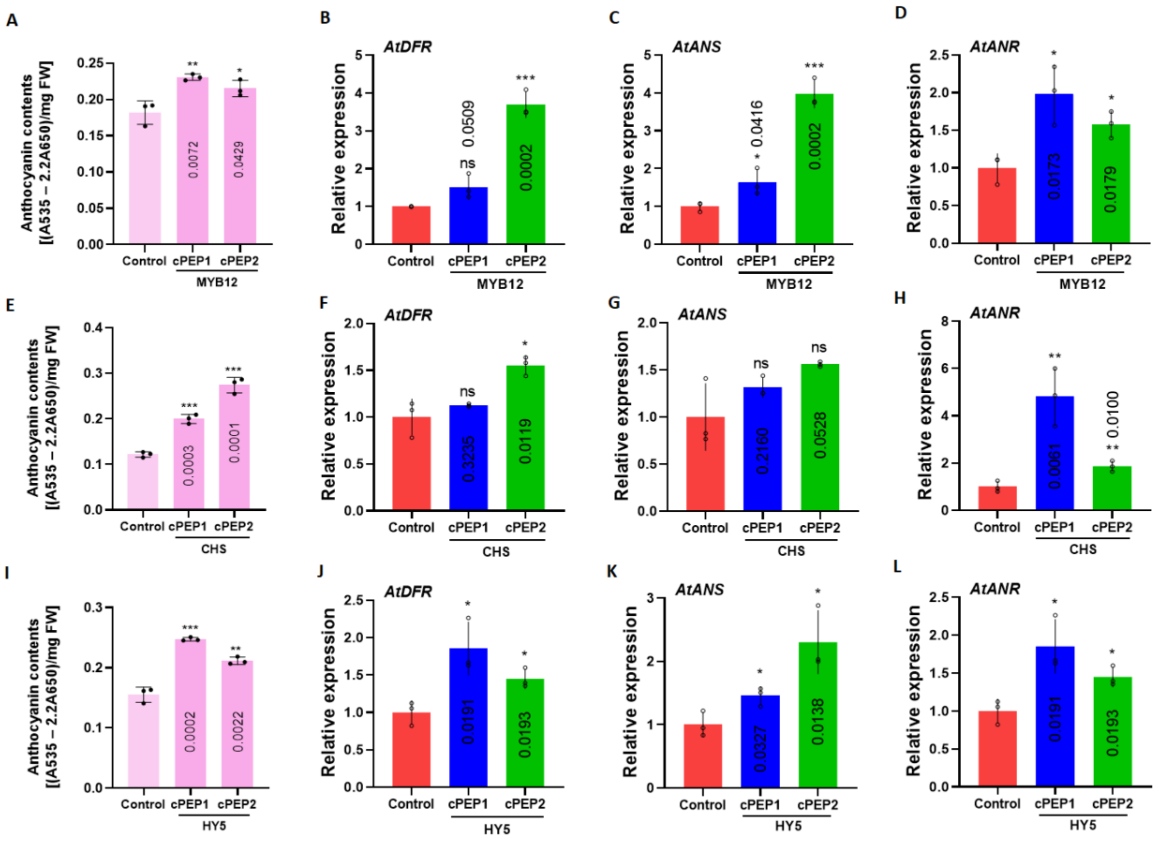

Supplemental Figure S12. Exogenous application of cPEPs (MYB12, CHS and HY5) enhances anthocyanin and its biosynthesis genes in Arabidopsis. (A, E, I) Quantification of anthocyanin in *Arabidopsis thaliana* seedlings grown on half-strength MS medium for 5-days and then dipped in liquid half-strength MS medium supplemented with water (control), and 0.50 µM cPEPs of MYB12, CHS, and HY5 for 48 hours. **(B, C, D, F, G, H, J, K, L)** Expression analysis of anthocyanin biosynthetic genes *AtDFR, AtANS and AtANR* in *Arabidopsis thaliana* seedlings grown on half-strength MS medium for 15-days and then dipped in liquid half-strength MS medium supplemented with water (control), and 0.50 µM cPEPs of MYB12, CHS, and HY5 for 48 hours.

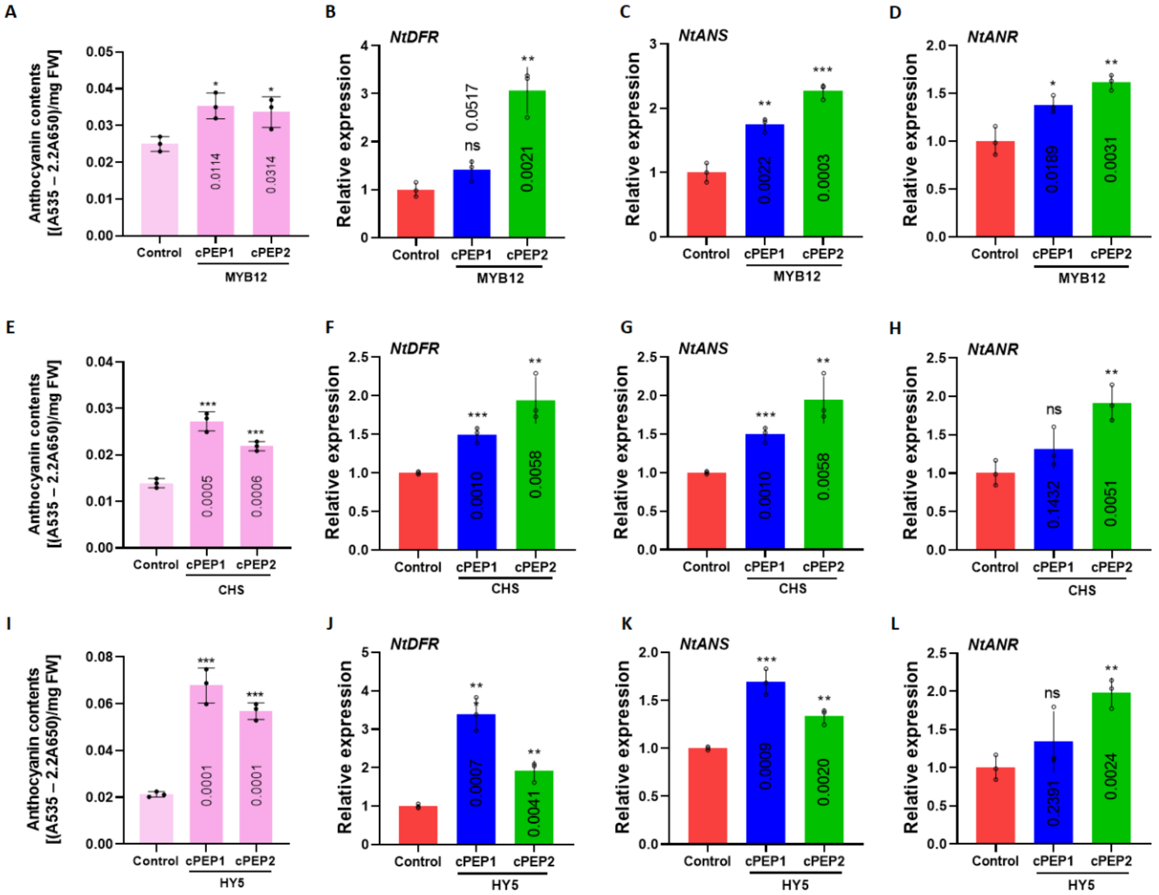

Supplemental Figure S13. Exogenous application of cPEPs (MYB12, CHS and HY5) enhanced anthocyanin and its biosynthesis genes in tobacco. (A, E, I) Quantification of anthocyanin in *Nicotiana tabacum* seedlings grown on half-strength MS medium for 15-days and then dipped in liquid half-strength MS medium supplemented with water (control), and 0.50 µM cPEPs of MYB12, CHS, and HY5 for 48 hours. **(B, C, D, F, G, H, J, K, L).** Expression analysis of anthocyanin biosynthetic genes *NtDFR, NtANS* and *NtANR* in *Nicotiana tabacum* seedlings grown on half-strength MS medium for 15-days and then dipped in liquid half-strength MS medium supplemented with water (control), and 0.50 µM cPEPs of MYB12, CHS, and HY5 for 48 hours.

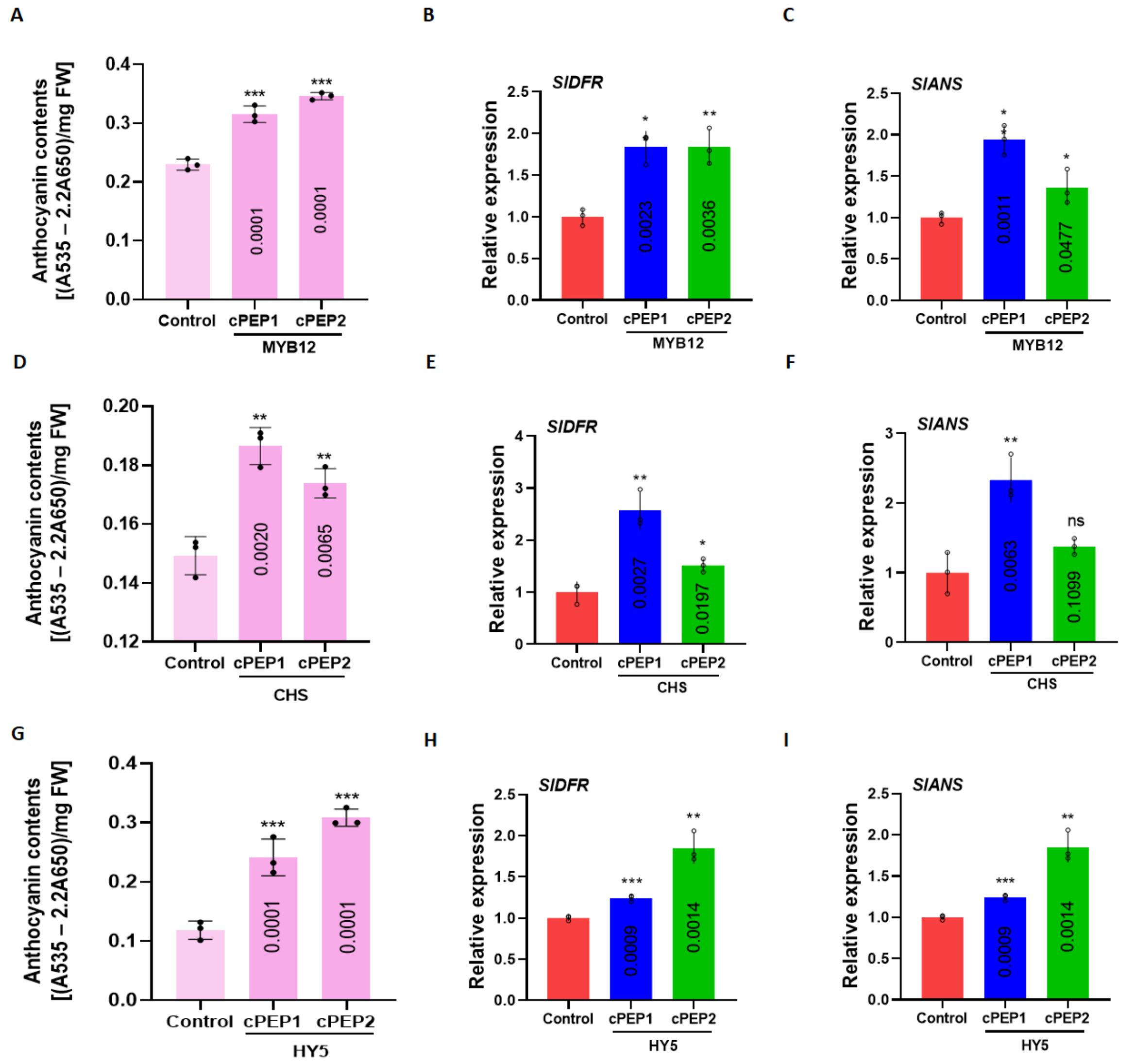

Supplemental Figure S14. Exogenous application of cPEPs (MYB12, CHS and HY5) enhances anthocyanin and its biosynthesis genes in tomato. (A, D, G) Quantification of anthocyanin in *Solanum lycopersicum* seedlings grown on half-strength MS medium for 10-days and then dipped in liquid half-strength MS medium supplemented with water (control), and 0.50 µM cPEPs of MYB12, CHS, and HY5 for 48 hours. (**B, C, E, F, H, I)** Expression analysis of anthocyanin biosynthetic genes *SlDFR* and *SlANS* in *Solanum lycopersicum* seedlings grown on half-strength MS medium for 10-days and then dipped in liquid half-strength MS medium supplemented with water (control), and 0.50 µM cPEPs of or MYB12, CHS, and HY5 for 48 hours.

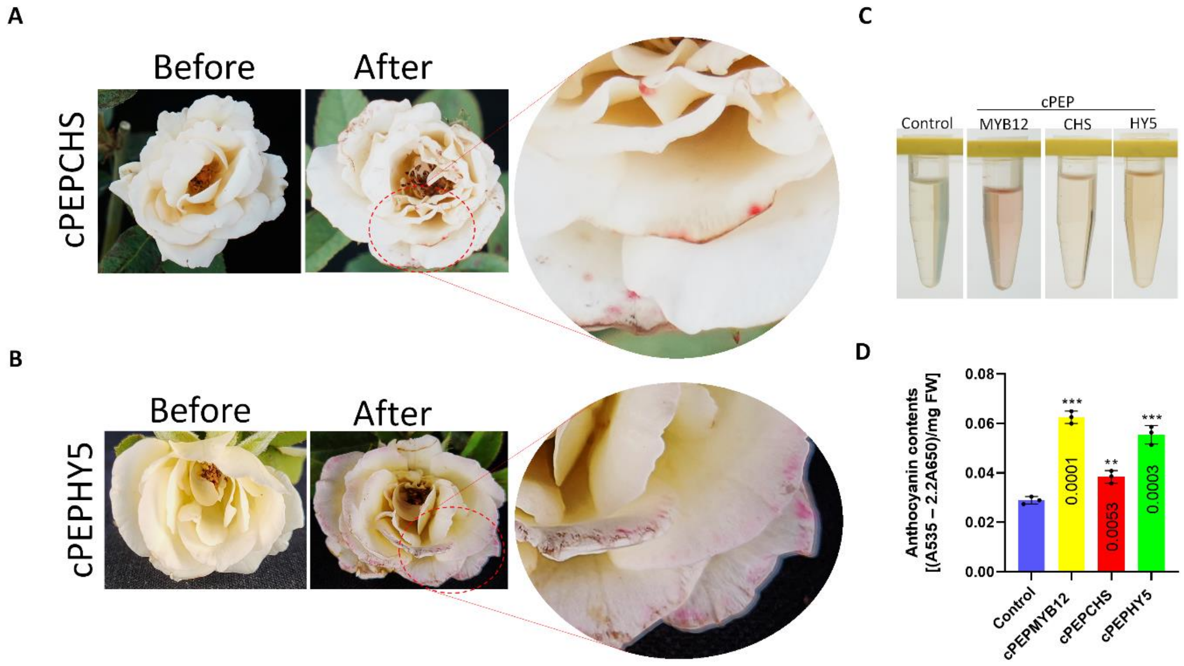

Supplementary Figure 15. Exogenous application of cPEP (CHS & HY5) induced colouring in white/yellow rose petals. (A, B) Representative image of rose before and after spray with cPEPCHS and cPEP HY5 respectively. **(C)** Representative image of anthocyanin accumulation in rose flower sprayed with water (control), 0.50 µM cPEPs (MYB12, CHS and HY5). **(D)** Quantification of anthocyanin in rose flower sprayed with half-strength liquid MS medium supplemented with water (control), 0.50 µM cPEPMYB12, cPEPCHS and cPEPHY5.

Table S1. List of primers used in the study

S.No.	Gene	Forward Primer (5' - 3')	Reverse Primer (5' - 3')
1	AtHY5	GGAGGAGAAGCTGTCGGAAAA	AATCTGGATCGGCGACCGG
2	AtMYB12	ACCAGGGAGAACAGACAACG	TCGTCATGATTACGGCGGAG
3	AtCHS	GGAGAAGTTCAAGCGCATGTG	ATGTGACGTTTCCGAATTGTCG
4	AtCHI	CTCTCTTACGGTTGCGTTTTCG	CACCGTTCTTCCCGATGATAGA
5	AtFLS	CCACCGTCATGCGTCAATTACAG	TCTCCGCCGAGACCTTCTTTCAA
6	AtF3H	TTCCTTACCTTCAGGCGGTTATC	CGAGAGTGGTGTTGGTGGATG
7	AtDFR	AGCCGCCAAGGGACGTTATATTTG	CCGGGAGAAAACCCTTTTGACGA
8	AtANS	GGCTGGTGTTTTGTGAGCCACCA	CCTTGGAGGAAACTTAGCCGGAGA
9	AtANR	AAGAAAACTGGACTGACGTTGAA	AACACCTTCGAGATTGGGTAAC
10	Tubulin	GAGCCTTACAACGCTACTCTGTCTGTC	ACACCAGACATAGTAGCAGAAATCAAG
11	GUS	CCTCGCATTACCCTTACGCTG	CTTGCTGAGTTTCCCCGTTG
12	NtHY5	GGCGGAGAAGCCACCGGAAC	TTCCTCTCCCTTGCTTGCTGTGCTG
13	NtMYB12	AGGTAGCAAACGTAGCCGC	TCTTGATCGGCAATGACCATT
14	NtCHS	CCTTTGGGAATTTCTGATTGG	TCCCACAATATAAGCCCAAGC
15	NtCHI	GACGGGTAAGCAATACTCAGAGAAG	AACTAGACTCCAATTTCTGGAATG
16	NtFLS	AAGTGATAAATCATGGAATTCCAG	TCTTCACCACCTGCTGCCTCCACT
17	NtF3H	AGCTAGAGACTACTCCAGGTG	AACCGTGATCCAAGTTTTGCCA
18	NtDFR	TCGAGTCCAAGGATCCTGAGAATG	CAACAACCAGCGGCGGTATGATG
19	NtANS	CCTACAGACTACATTCCAGCA	CCTTCTCTAGTCTTCCTTCTTCT
20	NtANR	GGAAATTTGCTGAGGAGAACA	GTGATCAAGGACATGGAAAGATT
21	SlHY5	GCAAGCGACGAGTTCTAT	ATCTCCGGCACTCTTCTG
22	SlMYB12	AAGTGGGCATCAAGAGAGGC	GGTAACGACCTCCAAGAGCC
23	SlCHS	TGGTCACCGTGGAGGAGTATC	GATCGTAGCTGGACCCTCTGC
24	SlCHI	GTTTTTCACAAACCAACAGTTCTGAT	GAAGCAGTGCTCGATTCCATAAT
25	SlFLS	GAGCATGAAGTTGGGCCAAT	TGGTGGGTTGGCCTCATTAA
26	SlF3H	CACACCGATCCAGGAACCAT	GCCCACCAACTTGGTCTTGTA
27	SlDFR	ATCGGCTCTTGGCTTGTCAT	ACAGCGTTAAGTTTGTATCAGC
28	SlANS	GAACTAGCACTTGGCGTCGAA	TTGCAAGCCAGGCACCATA
29	Actin	ATGACATGGAGAAGAATCTGGCATCA	AGCCTGGATGGCAACATACATAGC

Materials and Methods

Plant Materials. *Arabidopsis thaliana* (Col-0), *Nicotiana tabacum* cv. Pettit Havana, *Solanum lycopersicum* (micro tom) were used as wild type (WT) for various experiments in this study. Promoter: reporter lines (ProCHS::GUS, ProFLS::GUS, PromiR858:ORF::GUS) were developed earlier by our group, and used in this study for analysis (Sharma et al., 2020; Sharma et al., 2022; Bhatia et al., 2021). Arabidopsis mutants *myb12* (CS9602)*, hy5-215* (Oyama et al., 1997) were used in the study. The $NtHY5^{CR}$ and $SlHY5^{CR}$ plants developed using CRISPR/Cas9-based genome editing of HY5-encoding gene in tobacco and tomato, respectively, developed earlier by our group used in this study for analysis (Singh et al., 2024; Sinha et al., 2024). White and yellow roses were taken from CSIR-CIMAP, Lucknow Rose Garden. Grapes were purchased from the local market. The seeds were surface sterilized and placed on half-strength Murashige and Skoog medium (Hi-Media) with 1.5% sucrose, pH 5.72–5.8. After stratification for two days at 4°C in the dark, the plates were transferred to a growth chamber (Percival) with an ambient condition of 22°C temperature, 50–60% relative humidity, 180 $\mu molm^{-2}\ s^{-1}$ light intensity and 16-h-light/8-h-dark photoperiod cycle. Ten and fifteen-day-old seedlings were used for root length measurement for Arabidopsis and tobacco respectively. Five, fifteen-day-old seedlings were used for peptide assay and the relative expression analysis. For the DPBA staining experiment, three and seven-day-old seedlings grown on half-strength MS media were used for Arabidopsis, tobacco and tomato respectively.

Designing of cPEPs:

To craft the cPEPs, amino acid sequences of MYB12, CHS, and HY5 from *Arabidopsis thaliana, Nicotiana tabacum*, and *Solanum lycopersicum* was aligned. Ten amino acids from the conserved or aligned sequences were taken for designing cPEPs for each gene (Supplemental Figure S1).

Peptide assay. The synthetic peptides (purity>95%) were synthesized through Link Biotech (http://www.linkbiotech.com). The peptides were dissolved in milliQ water (stock concentration, 10 mM). For expression analysis, western blot, flavonoid and anthocyanin quantification and promoter reporter experiment, seeds were grown in half-strength MS media for five or ten-days and then dipped in liquid half-strength MS medium supplemented with peptide at a concentration of 50 µM and liquid half-strength MS medium with MQ were used as control. For DPBA staining, seeds were placed in half-strength MS media supplemented with 50 µM concentration of synthetic peptides. For rose (complete flower), the synthetic

peptides were dissolved in Milli-Q water at a concentration of 50 µM and sprayed on them. For grapes fruit were plucked from pedicel to enhanced maximum absorption of peptide.

Peptide sequences:

cPEP1MYB12: GKSCRLRWVN (10 aa)
cPEP2MYB12: GRTDNDVKNF (10 aa)
cPEP1CHS: RQDIVVVEVP (10 aa)
cPEP2CHS: GARVLVVCSE (10 aa)
cPEP1HY5: KENKRLKRLL (10 aa)
cPEP2HY5: QARERKKAYL (10 aa)

Expression analysis. For expression analysis, total RNA was isolated using Plant total RNA kit (Sigma), and was treated with DNase (Thermo); 1 µg of total RNA was reverse transcribed using Revert AidH Minus First Strand cDNA Synthesis Kit (Thermo) to produce cDNA. This cDNA was diluted nearly 20 times using nuclease-free water and 2 µl of this diluted cDNA was used as template for quantitative Real- Time PCR done using Fast SYBR Green Mix (Applied Biosystems) in a Fast 7500 Thermal Cycler instrument (Applied Biosystems). The expression was normalized using Tubulin (in case of Arabidopsis) or Actin (in case of tomato and tobacco) and comparative expression was calculated through the comparative $^{\Delta\Delta}$CT method (Livak and Schmittgen 2001). The primer sequences used for the expression analysis are listed in Supplementary Table 1.

Total protein extraction and western blot analysis. For protein extraction and western blot analysis, the experiments were done as per the methods described in Sharma et al. (2020). The western blot images were captured by Image Lab version 5.2.1 build 11 (Bio-Rad Laboratories). Commercial antibodies anti-Actin (A0480, Sigma Aldrich), anti-CHS (AS122615, Agrisera), anti-MYB12 (Eurogenetec) and anti-HY5 (Agrisera) were used in the analysis.

Lignin accumulation staining. For lignin staining, the roots of 10-day-old seedlings were stained with basic fuchsin (Sigma-Aldrich) as per the method in the ClearSee protocol described by Ursache *et al.* 2018. The images of stained roots were captured in LSM880-Airy scan confocal microscope at 561 nm excitation and detection was done at 600-650 nm to visualize lignin deposition in the roots.

Histochemical GUS staining. The histochemical GUS staining was done using a method previously described by Jefferson 1989. 5-day old seedlings of *A. thaliana* were immersed in GUS solution containing 100-mM sodium phosphate buffer (pH 7.2), 10- mM EDTA, 0.1% Triton X-100, 2-mM potassium ferricyanide, 2-mM potassium ferrocyanide, and 1 mg mL^{-1} 5-bromo-4- chloro-3-indolyl-b-D-glucuronide and incubated at 37^0C for 4 h. The seedlings were then given multiple washes with 70% ethanol to remove the chlorophyll and imaged for GUS under a Leica microscope (LAS version 4.12.0; Leica Microsystems, Wetzlar, Germany).

Total anthocyanin quantification. Total anthocyanin was estimated according to the method previously described by Sharma *et al.* 2020 with slight modifications. About 300 mg of seedlings treated with or without cPEPs were crushed in liquid N_2, transferred into 500 µl anthocyanin extraction buffer (Propanol:HCl: H_2O::18:1:81), and incubated at 4^0C overnight under shaking conditions. The samples were centrifuged at 12,000 g at 4^0C for 15 minutes. The absorbance of the supernatant was measured at A_{535} and A_{650} and the total anthocyanin was quantified using the formula (A535−2.2A650)/g FW.

Extraction and quantification of flavonols. For the extraction of flavonols, the 500 mg of seedlings treated with or without cPEPs, were crushed in liquid N_2 and extracted in 80% methanol overnight at 4^0C. The extracts were hydrolyzed in an equal amount of 6N HCl at 70°C for 40 min followed by the addition of an equal amount of methanol to prevent the precipitation of the aglycones. The extracts were filtered through 0.22 µm filters (Millipore) and the quantification of various metabolites of the samples was done by HPLC–PDA using a Waters 1525 Binary HPLC Pump system comprising PDA detector following the method described by Sharma *et al.* 2020. The calculation was done by Breeze 2 software (Waters).

DPBA Staining. For DPBA staining, three and seven-day-old seedlings were stained in 1 ml DPBA solution containing 0.25% diphenyl boric acid β-aminoethyl ester (DPBA) and 0.01% Triton X-100 by shaking for 15 min at room temperature under dark condition. The staining solution was removed and the seedlings were washed with autoclaved distilled water for 5 min about 2-3 times. The fluorescence of DPBA- kaempferol and DPBA-quercetin was visualized on a LSM880-Airy scan confocal microscope. The images of stained roots were captured by confocal microscope at 475-500 nm (for kaempferol) and 585-619 nm (for quercetin) estimation.

Statistical analysis. The statistical tests and n numbers, including sample sizes or biological replications, are described in the figure legends. All the statistical analyses were performed

using two-tailed Student's t-tests using GraphPad Prism version 9.5.1. software. Experimental details regarding biological or technical replicates and statistical parameters used in various experiments are provided in Methods and also in the figures and supplementary figures legend, wherever necessary.

Bhatia C, Gaddam SR, Pandey A, Trivedi PK (2021) COP1 mediates light-dependent regulation of flavonol biosynthesis through HY5 in Arabidopsis. Plant Sci **303:** 110760.

Oyama T, Shimura Y, Okada K (1997) The Arabidopsis HY5 gene encodes a bZIP protein that regulates stimulus-induced development of root and hypocotyl. Genes Dev. **11:** 2983-2995.

Livak KJ, Schmittgen TD (2001) Analysis of relative gene expression data using real-time quantitative PCR and the 2 $-\Delta\Delta^{CT}$ method. Methods **25:** 402–408.

Ursache R, Andersen TG, Marhavý P, Geldner N (2018) A protocol for combining fluorescent proteins with histological stains for diverse cell wall components. Plant J **93:** 399-412.

Jeferson RA (1989) GUS reporter gene system. Nature **342:** 837–838.

Singh D, Dwivedi S, Sinha H, Singh N, Trivedi PK (2024) Mutation in shoot-to-root mobile transcription factor, ELONGATED HYPOCOTYL 5, leads to low nicotine levels in tobacco. J Hazard Mater **465:** 133255.

Sinha H, Kumar RS, Datta T, Singh D, Srivastava S, Trivedi PK (2024) Steroidal glycoalkaloid biosynthesis and fungal tolerance are regulated by ELONGATED HYPOCOTYL 5, SlHY5, in tomato. Plant Physiology (Accepted contingent on revision).

Sharma A, Badola PK, Gautam H, Gaddam, S.R. Trivedi PK (2022) HY5 regulates light-dependent expression and accumulation of miR858a-encoded peptide, miPEP858a. Biochem Biophys Res Commun **589:** 204-208
